# Items

**Published:** 1901-03

**Authors:** 


### ITEMS.

THE Maltine Company has distributed with its compliments a Physician's Visiting List, which is a pocket memorandum-book of great convenience. It is bound in black seal, flexible leather, is of convenient size, made of good paper, with gilt edges and rounded corners and is a ready reminder of the many excellent preparations made in the laboratories of this solid house.

---

THE Mellier Drug Company, 2112 Locust Street, St. Louis, Mo., on receipt of $1.00 will send, charges prepaid, a half-minute, self-registering clinical thermometer, with magnifying index and certificate of registration, in a handsome and durable aluminum case with chain and pin attachment. In addition it will send samples of tongaline liquid, tongaline and lithia tablets, tongaline tablets, tongaline and quinine tablets and ponca compound. Remit currency, money order, exchange on St. Louis, Chicago or New York—no personal check.

